# Supplementary material for: Motor cortex somatostatin interneurons adaptively shape the structure of action sequences
Source: Nat Commun. 2026 Mar 18;17:4116. doi: 10.1038/s41467-026-70353-y (PMC13149988; doi:10.1038/s41467-026-70353-y)
Supplement: Supplementary file 1 — Supplementary information [file 41467_2026_70353_MOESM1_ESM.pdf]

Supplementary Materials

Motor cortex somatostatin interneurons adaptively shape the structure of action sequences

Jeong Oen Lee[1], Sebastiano Bariselli[1,2,3], Giacomo Sitzia[1,4], Abigail Holder[1], and David M. Lovinger[1+]

1. Laboratory for Integrative Neuroscience (LIN), National Institute on Alcohol Abuse and Alcoholism, Bethesda, MD, USA
2. Current address: IRCCS Humanitas Research Hospital, Via Manzoni 56, 20089 Rozzano, Milano, Italy
3. Current address: Department of Biomedical Sciences, Humanitas University, Via Rita Levi Montalcini 4, 20072 Pieve Emanuele, Milan, Italy
4. Current address: Department of Neuroscience, Københavns Universitet, København, Hovedstaden, Denmark

+Lead corresponding author: David M. Lovinger, lovindav@mail.nih.gov

Supplementary Figure1

Supplementary Figure2

Supplementary Figure3

Supplementary Figure4

Supplementary Figure5

Supplementary Figure6

Supplementary Figure7

Supplementary Figure8

Supplementary Figure9

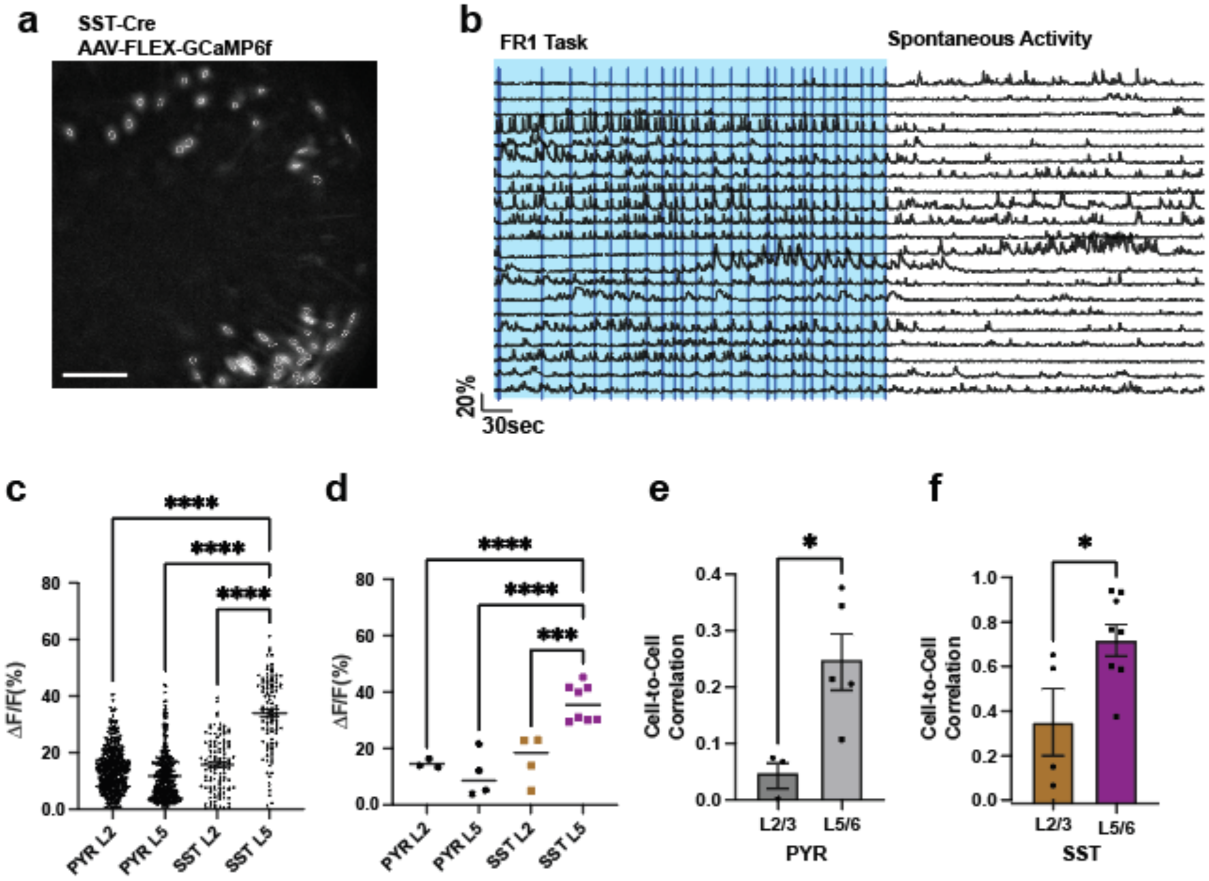

Supplementary Figure 1. Action-related SST activity during the FR1 task diminished after the training session.
(a) Example image displaying the maximum $\Delta F/F_0$ projection during the training session, with white circles indicating contours of identified neurons, scale bar: 200um. **(b)** Representative calcium traces during and after the FR1 task. The blue shaded area indicates the FR1 training period, while the blue vertical bars indicate the lever presses. The lever was retracted after the FR1 task finished, and each trace represent the activity of same neuron. **(c)** Average $\Delta F/F_0$ responses of individual PYR neurons (n=484 in L2/3 and n= 397 in L5/6) and SST INs in L2/3 (n=116) and L5/6 (n=83) aligned to lever press (Kruskal–Wallis test, main effect $p < 0.0001$). **(d)** Population-averaged $\Delta F/F_0$ responses across individual mice (Ordinary one-way ANOVA; PYR L2/3 n=6 mice, PYR L5/6 n=4 mice, SST L2/3 n=4 mice, SST L5/6 n=8 mice; main effect $p < 0.0001$). **(e)** Cell-to-cell correlation coefficients of PYR neurons in L2/3 (n=3 mice) and L5/6 (n=5 mice), each dot denotes an animal measurement on D7 (two-tailed unpaired t-test, *p=0.0357). **(f)** Cell-to-cell correlation coefficient of SST INs in L2/3 (n=4 mice) and L5/6 (n=8 mice), each dot denotes an animal measurement on D7 (two-tailed unpaired t-test, *p=0.0281).

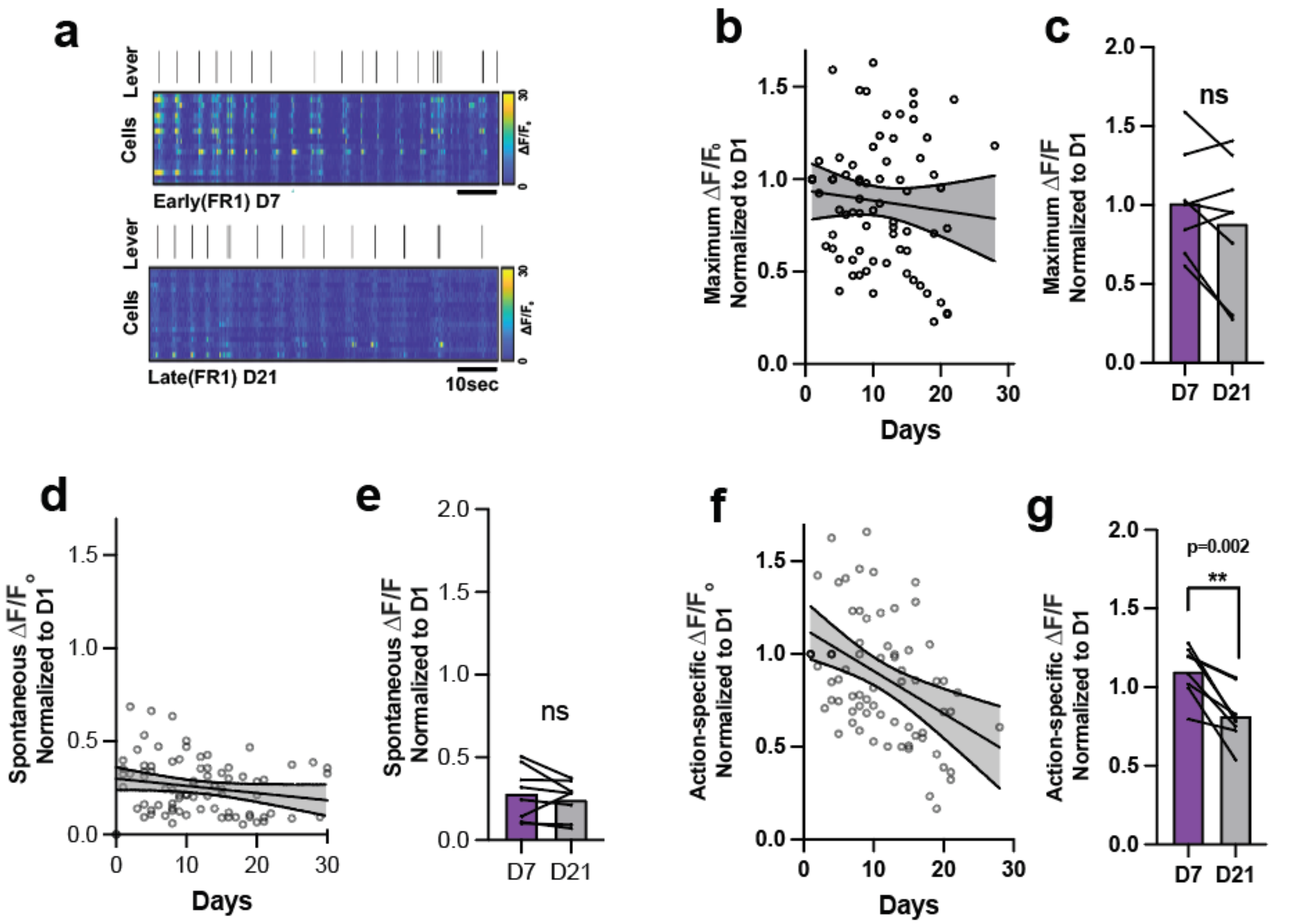

Supplementary Figure 2. Action-locked SST activity in M1 L5/6 undergoes changes with motor training.
(a) Example $\Delta F/F_0$ population activity map of SST interneurons within a subject on D7 and D21 during an FR1 training session, with vertical bars indicating lever presses. **(b)** Maximum $\Delta F/F_0$ response of L5/6 SST normalized to Day1 measurement. Fluorescence degradation was not significant (linear regression R^2=0.0095, slope non-zero p=0.7038) indicating consistent imaging measurement over 21 days. Each dot indicates max $\Delta F/F_0$ of one training session, and **(c)** comparison of the maximum $\Delta F/F_0$ ratio between D7 and D21 (n=8 mice, two-tailed paired t-test, p =0.1276). **(d)** Spontaneous background $\Delta F/F_0$ over 21 days. **(e)** No statistical difference between D7 and D21 in the amplitude of spontaneous $\Delta F/F_0$ (n=8 mice, two-tailed paired t-test, p =0.1276). **(f)** Action-locked $\Delta F/F_0$ response of L5/6 SST normalized to Day1 measurement. Response degradation was significant (linear regression R^2=0.1595, slope non-zero p=0.0004). Each dot indicates average $\Delta F/F_0$ time-locked to lever-press within a training session, and **(g)** Action-locked $\Delta F/F_0$ ratio significantly decreased from D7 to D21 (n=8 mice, two-tailed paired t-test, p =0.0020). In all plots, shaded area and error bars denote SEM.

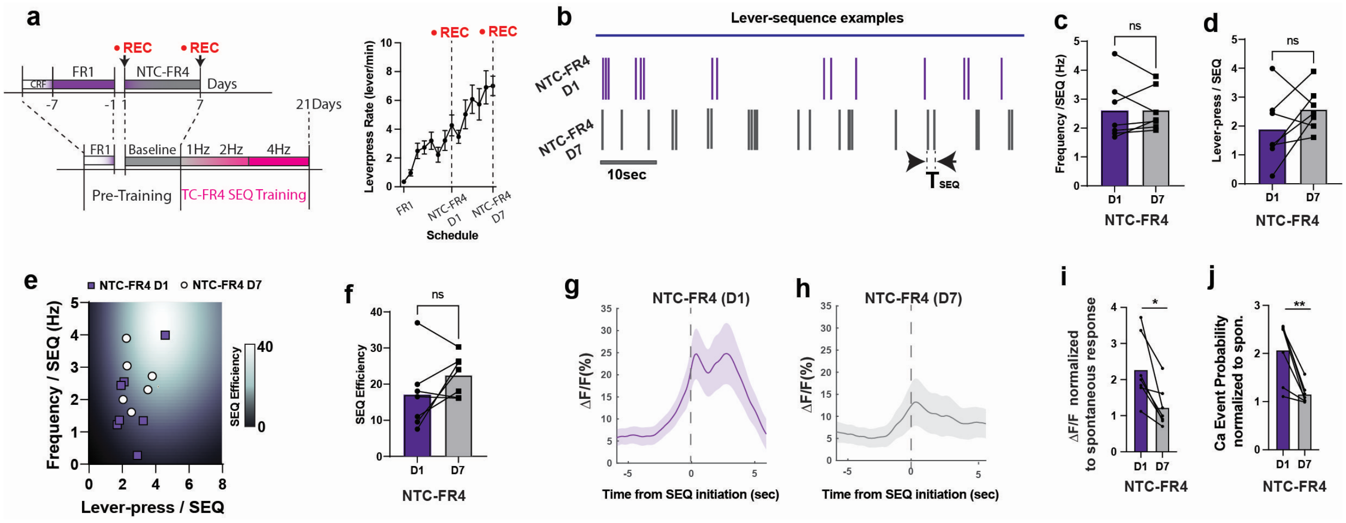

Supplementary Figure 3. SST-IN activity in M1 L5/6 was stabilized while the similar action sequence structure was maintained.
(a) Pre-training schedule including FR1 and non-time-constrained FR4 training, before starting time-constrained sequence lever-press training. Average lever-press frequency per session (lever/min) increased as expected. In this static training schedule, consecutive lever-press and time-limit were not assessed for the reward delivery. **(b)** Example lever-press patterns on the first day (D1, upper panel) and the seventh day (D7, lower panel) of non-time-constrained FR4 training. **(c,d)** lever-press frequency and lever-press number per a unit sequence trial were not significantly altered for the 7 days of non-time-constrained FR4 training (n= 7mice, two-tailed paired t-test, p=0.9524, p= 0.1852), despite the increase in overall leverpress rate. Thus, the action kinematics of the action sequence was maintained. **(e)** Leve-press number and frequency (Hz) are projected onto the sequence efficiency coefficient map in D1 (circle) and D7(square) of non-time-constrained FR4 training. **(f)** The sequence efficiency coefficients between the D1 and D7 were not altered. (n= 7 mice, two-tailed paired t-test, p=0.1680). **(g)** averaged $\Delta F/F_0$ (%, F_0 is spontaneous baseline) of SST interneurons in the non-time-constrained FR4 training schedule(D1), and **(h)** for D7. **(i)** action-specific $\Delta F/F_0$ peak in each animal, normalized to the spontaneous $\Delta F/F_0$ peak response, comparison between D1 and D7 (n=7 mice, two-tailed paired t-test *p =0.010) **(j)** action-specific $\Delta F/F_0$ peak in each animal, normalized to the spontaneous $\Delta F/F_0$ peak response, comparison between D1 and D7 (n= 7mice, two-tailed paired t-test, **p=0.0041). In all plots, shaded area and error bars denote SEM.

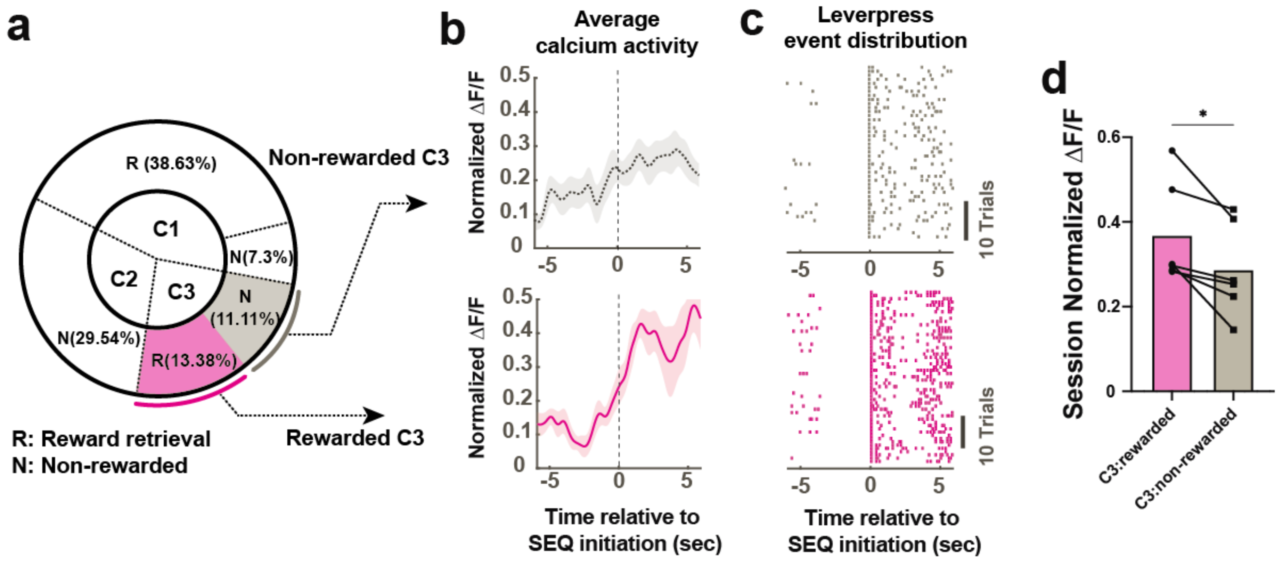

Supplementary Figure 4. Sub-classes of rewarded and non-rewarded SEQs involve different SST-IN activity
(a) Rewarded and non-rewarded sequences within C3. **(b)** Average fluorescence traces of SST population activity corresponding to non-rewarded (upper) and rewarded sequences (bottom) within C3, normalized to session maximum and time-locked to the sequence initiation. **(c)** Example lever-press distribution of non-rewarded sequences (upper) and (j) the same for rewarded sequences (bottom) within C3. **(d)** Maximum ΔF/F calcium responses (between 0-5 sec) normalized within a session between C3 rewarded SEQ and C3 non-rewarded SEQs. (two-tailed paired sample Wilcoxon test, n=6 mice) *p=0.0313). In all plots, shaded area and error bars denote SEM.

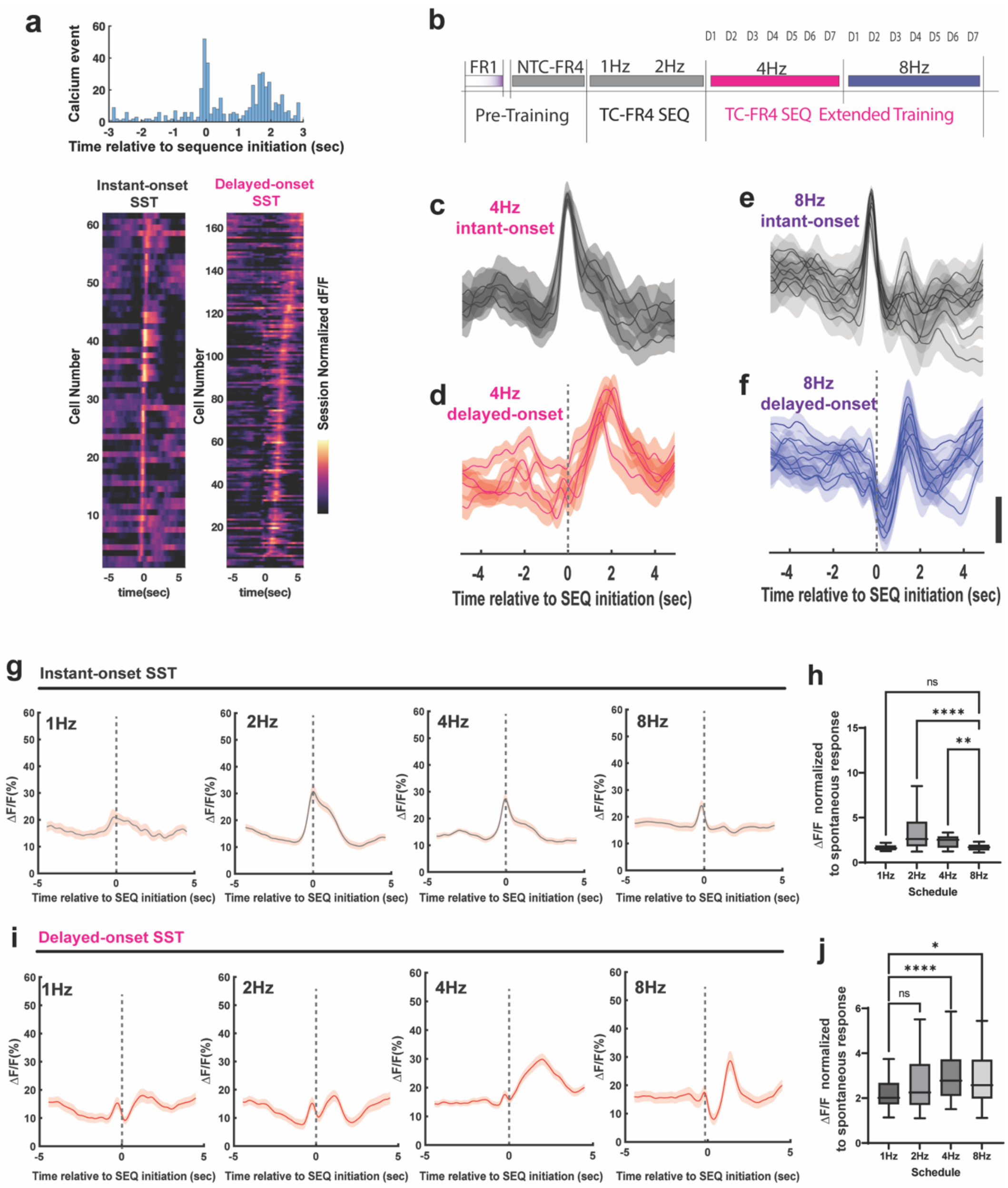

Supplementary Figure 5. Subpopulations of SST INs continuously activated during the execution of complex action sequences

(a) Bimodal distribution of SST IN calcium events during TC-FR4SEQ training (histogram generated from 712 averaged calcium profiles). Representative color coded-map of single cell calcium responses (session normalized $\Delta F/F_0$) for 'instant-onset SST' (n=62 cells, 3mice; bottom left) and 'delayed-onset SST' (n=163 cells, 3mice; bottom right), color values represent the session normalized $\Delta F/F_0$ of each SST IN, averaged over a training session. **(b)** Measurement schedule of the extended TC-FR4 SEQ training with 4Hz and 8Hz schedules. **(c)** Average calcium profiles of 'instant-onset SST' in the 4Hz schedule, with each trace representing the daily average of session normalized $\Delta F/F_0$ (n=3mice). Measurements were performed for 4 consecutive days. **(d)** Average calcium profiles of 'delayed-onset SST' concurrently measured during the same 4Hz schedule. **(e)** Average calcium profiles of 'instant-onset SST' in the 8Hz schedule, with each trace representing the daily average of session normalized $\Delta F/F_0$ (n=3mice). Measurements were performed for 7 consecutive days. **(f)** Average calcium profiles of 'delayed-onset SST' concurrently measured during the same 8Hz schedule. The vertical bar represents a value of 0.1 in the session-normalized ΔF/F0. **(g)** Average calcium profiles ($\Delta F/F_0$,%) of 'instant-onset SST' in TC-FR4 SEQ 1Hz, 2Hz, 4Hz and 8Hz schedules. **(h)** Peak $\Delta F/F_0$ to spontaneous calcium response calculated within each cell (Kruskal-Wallis test, p<0.0001; Dunn's multiple comparisons, 8Hz vs 2Hz p<0.0001, 8Hz vs 4Hz p=0.0024, 8Hz vs 1Hz p>0.9). Under the TC-FR4 8Hz schedule, single cell calcium modulation was significantly lower compared to the 2Hz and 4Hz schedules (1Hz n=9 cells, 2Hz n=39 cells, 4Hz n=36 cells, 8Hz n=35 cells). **(i)** Average calcium profiles ($\Delta F/F_0$,%) of 'delayed-onset SST' in TC-FR4 SEQ 1Hz, 2Hz, 4Hz and 8Hz schedules. **(j)** Peak $\Delta F/F_0$ to spontaneous calcium response calculated within each cell (Kruskal-Wallis test, p=0.0002; Dunn's multiple comparisons, 1Hz vs 2Hz p=0.4086,1Hz vs 4Hz p<0.0001, 1Hz vs 8Hz p=0.03). Under the 4 and 8Hz schedules, single cell calcium modulation was significantly higher compared to the 1Hz schedule (1Hz n=58 cells, 2Hz n=62 cells, 4Hz n=115 cells, 8Hz n=59 cells).

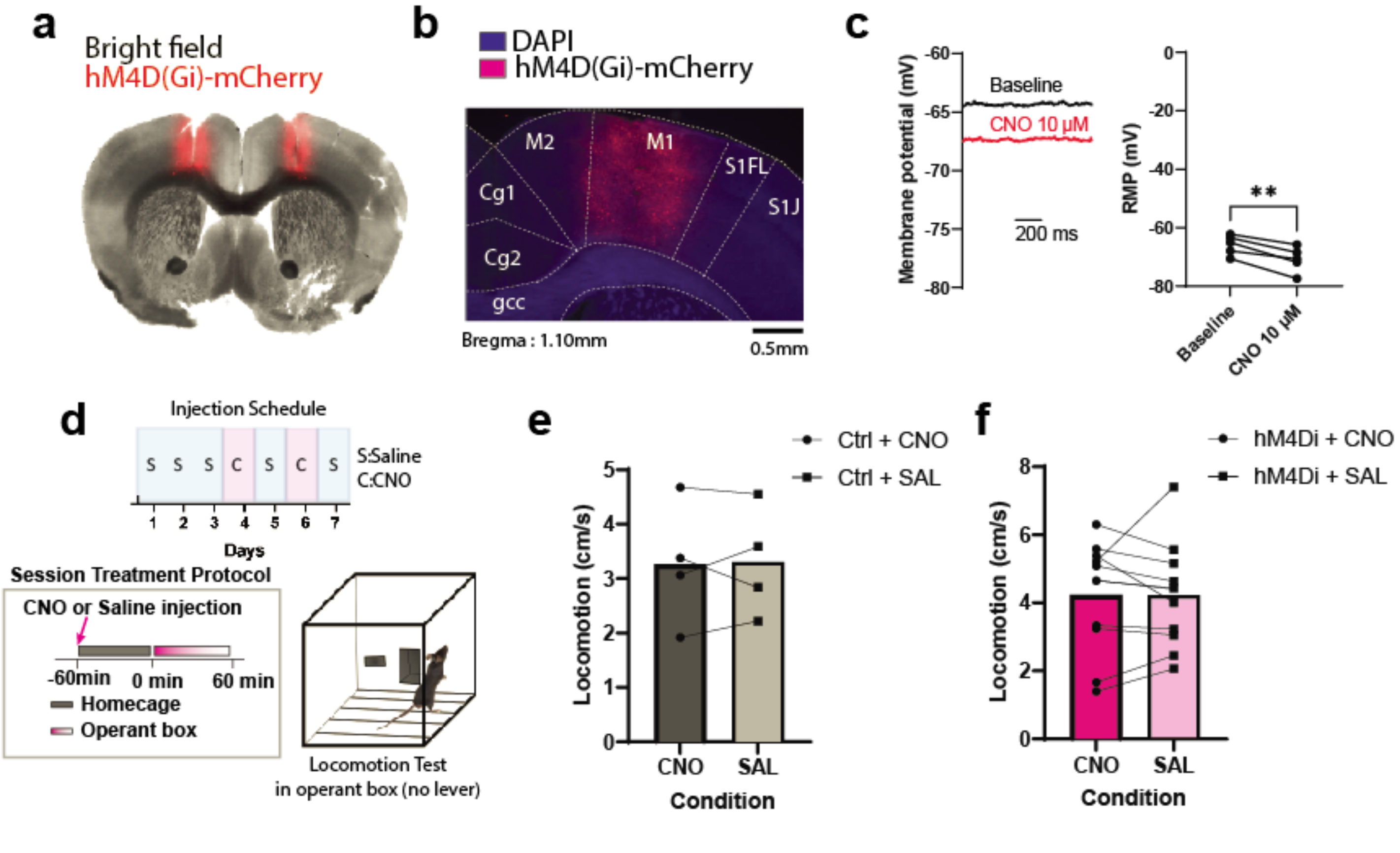

Supplementary Figure 6. hM4Di and CNO application alters resting membrane potential of SST interneurons in brain slices, with no significant change during measurement of locomotion in the operant chamber in the absence of a lever. (a) An example of a brain slice image used in electrophysiology experiments showing the expression of hM4Di-mCherry in M1 SST neurons. Slice thickness: 300µm. **(b)** Fluorescence image displaying hM4Di-mCherry expression in M1 SST neurons. **(c)** Effects of CNO (10µM) bath application on resting membrane potential (mV) measured in hM4Di-mCherry M1 SST neurons (N = 5 cells, 5 slices, 3 mice) (paired t-test, two-tailed, $p^{**} = 0.0036$). **(d)** Diagram of the locomotion test in the operant box and injection schedule. **(e)** Control mice (SST Cre-negative, n=4) exhibited no significant locomotion change between CNO and saline injection, as expected (two-tailed paired t-test p=0.877). **(f)** hM4Di-DREADD expressing mice (SST Cre-positive, n=11) did not demonstrate difference in locomotion between CNO and saline injection (two-tailed paired t-test p=0.973). These mice are a separate cohort from the TC -FR4 training experiment in Fig.5, with no prior sequence training or injection history.

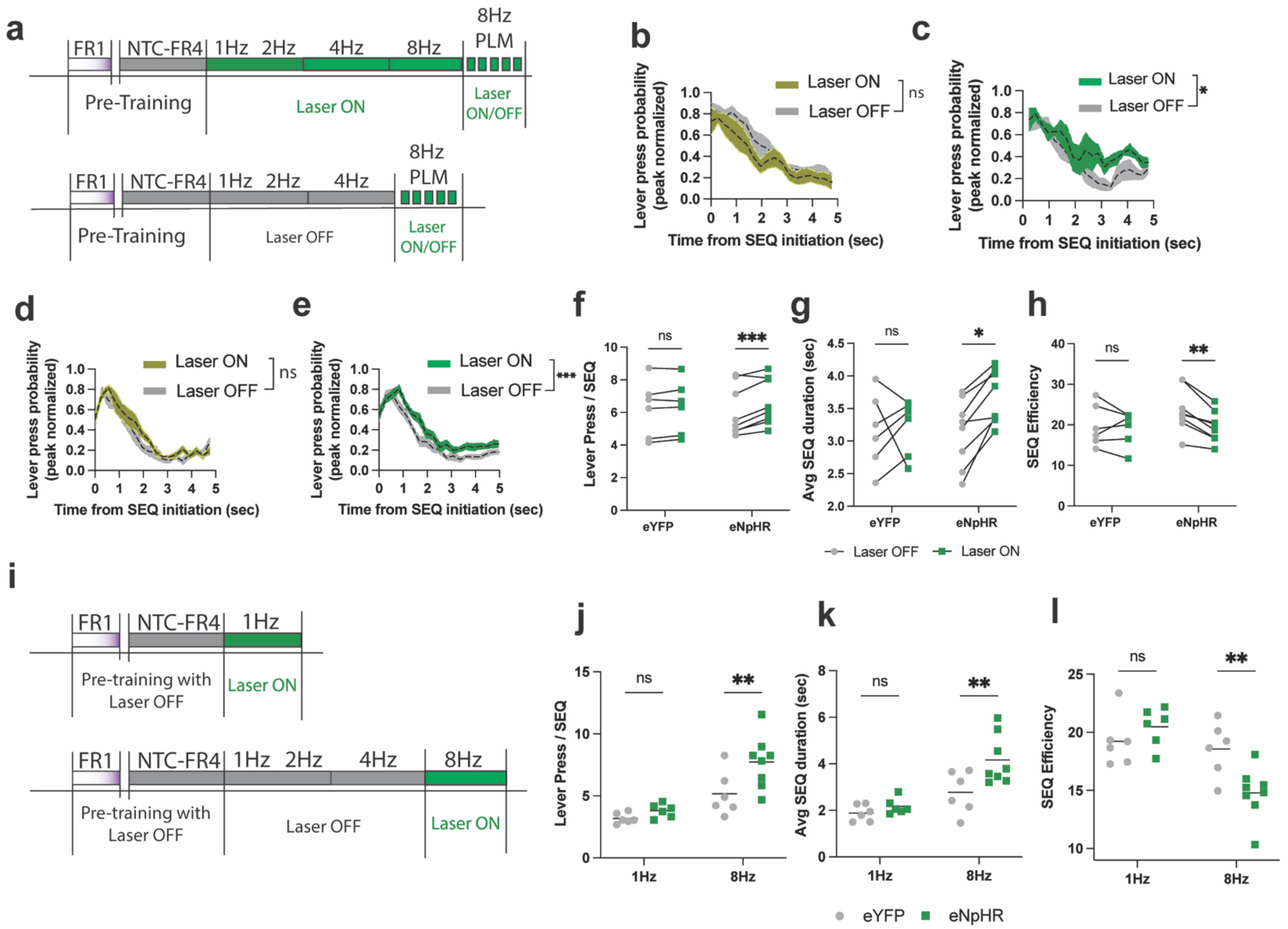

Supplementary Figure 7. Lever-press distributions are actively modulated by laser ON/OFF controls, and immediate optogenetic inactivation effects of SST-INs differ depending on task. (a) TC-FR4 sequence training combined with the PLM schedule performed after long-term optogenetic suppression (top) and TC-FR4 sequence training conducted without prior optogenetic inactivation (bottom). **(b)** Average peak-normalized lever-press probability distribution after long-term optogenetic supersession. Time = 0 corresponds to sequence initiation (SEQ initiation press excluded). The lever-press probability distribution was unchanged in the eYFP group with laser ON/OFF modulation (two-way ANOVA: Time, ****p < 0.0001; Laser ON/OFF, p = 0.1505; Time × Laser ON/OFF, p = 0.6518). **(c)** Average peak-normalized lever-press probability distribution aligned to sequence initiation (SEQ initiation press excluded) after long-term optogenetic supersession. The eNpHR group exhibited significant difference in lever-press probability distribution depending on laser ON/OFF modulation (two-way ANOVA: Time, **p = 0.0042; Laser ON/OFF, *p = 0.012; Time × Laser ON/OFF, p = 0.5317). **(d)** Average peak-normalized lever-press probability distribution across five sessions (30 min each) conducted without prior optogenetic inactivation. Time = 0 corresponds to sequence initiation (SEQ initiation press excluded). The eYFP group showed no significant difference between laser ON and OFF trials (two-way ANOVA: Time, p < 0.0001; Laser ON/OFF, p = 0.0950; Time × Laser ON/OFF, p = 0.2752). **(e)** Average peak-normalized lever-press probability distribution across five sessions (30 min each) conducted without prior optogenetic inactivation. Time = 0 corresponds to sequence initiation (SEQ initiation press excluded). The eNpHR group showed an elongated lever-press probability distribution on laser ON trials compared to laser OFF trials (two-way ANOVA: Time, ****p < 0.0001; Laser ON/OFF, ***p = 0.0009; Time × Laser ON/OFF, p = 0.0433). **(f)** Lever-presses per sequence (SEQ) (two-way repeated measure ANOVA: Group, p=0.9242; Laser, **p = 0.0018; Group × Laser, *p =0.0192; Sidak's multiple comparisons test: eYFP, p = 0.6576; eNpHR, ***p = 0.0005). **(g)** Average SEQ duration (two-way repeated measure ANOVA: Group, p=0.3705; Laser, *p = 0.0341; Group × Laser, p =0.0715; Sidak's multiple comparisons test: eYFP, p = 0.9555; eNpHR, *p = 0.0118). **(h)** SEQ efficiency (two-way repeated measure ANOVA: Group, p=0.4145; Laser, **p = 0.0080; Group × Laser, *p =0.0429; Sidak's multiple comparisons test: eYFP, p = 0.8049; eNpHR, **p = 0.0027). **(i)** TC-FR4 sequence training without prior optogenetic inactivation, the laser activation was enabled at 1Hz schedule (top) and 8Hz schedule (bottom). **(j)** Lever-presses per sequence (SEQ) showed non-significant Group × Schedule interaction and a main effect of Schedule (two-way ANOVA: Group, ***p <0.0001; Schedule, *p = 0.0140; Group × Schedule, p =0.1160; Sidak's multiple comparisons test: 1Hz, p = 0.7360; 8Hz, **p = 0.0092). **(k)** Average SEQ duration (s) showed a significant Group × Schedule interaction and main effects of both factors (two-way ANOVA: Group, ***p =0.0001; Schedule, *p = 0.0124; Group × Schedule, p =0.0848; Sidak's multiple comparisons test: 1Hz, p = 0.7841; 8Hz, **p = 0.0063). **(l)** SEQ efficiency also showed significant main effects and interaction (two-way ANOVA: Group, **p =0.0010; Schedule, p = 0.1479; Group × Schedule, **p =0.0066; Sidak's multiple comparisons test: 1Hz, p = 0.5310; 8Hz, **p = 0.0066). 1Hz eYFP n=6 mice, 1Hz eNpHR n= 6mice, 8Hz eYFP n=6 mice, 8Hz eNpHR n=8 mice.

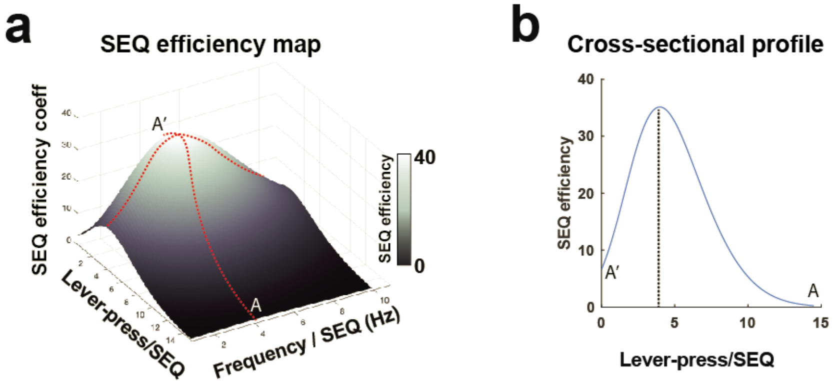

Supplementary Figure 8. SEQ efficiency map can be parameterized using sequence-related variables. (a) A color-coded surface profile illustrating the SEQ efficiency map generated by skewed gaussian functions. **(b)** A cross-sectional profile of the SEQ efficiency map along the red line, spanning from A-A', with the frequency/SEQ set at 4Hz. The peak locations in the color-coded surface and the distribution curve indicate the most optimal structure of action sequences with 4Hz and 4 lever-presses per SEQ.

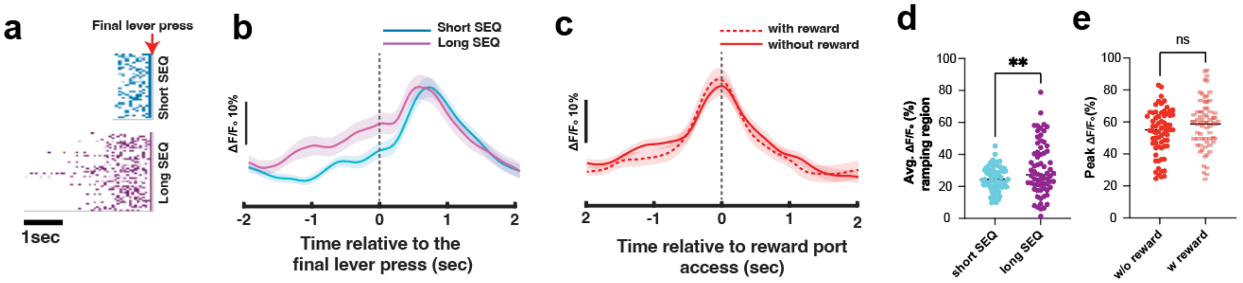

Supplementary Figure 9. Delayed onset SST INs profile reflects ongoing action sequence structure and subsequent transition. (a) Examples of lever press event aligned to the final lever press of each sequence during TC-FR4 4Hz training. **(b)** Prior the final lever press (time 0), calcium profiles of delayed-onset SST INs exhibit distinct ramping patterns from −2 to 0 seconds between short SEQ and long SEQ, but a calcium peak is observed following the final lever press. **(c)** This calcium peak is associated with reward port access and is not influenced by the presence or absence of reward. **(d)** Average calcium activity of delayed-onset SST INs prior to the final lever press (ramping region from −2 to 0 seconds) varies significantly with sequence structure (two tailed unpaired t-test, delayed onset SST, n= 68 cells, **p=0.0059). **(e)** Calcium peak amplitude around reward port access does not significantly differ based on reward outcome (two tailed unpaired t-test, delayed onset SST, n= 68 cells, p=0.1109).
