# Supplementary material for: Motor cortex somatostatin interneurons adaptively shape the structure of action sequences
Source: Nat Commun. 2026 Mar 18;17:4116. doi: 10.1038/s41467-026-70353-y (PMC13149988; doi:10.1038/s41467-026-70353-y)
Supplement: Supplementary file 2 — Reporting Summary [file 41467_2026_70353_MOESM2_ESM.pdf]

nature portfolio

Corresponding author(s): David Lovinger

Last updated by author(s): Oct 17, 2024

Reporting Summary

Nature Portfolio wishes to improve the reproducibility of the work that we publish. This form provides structure for consistency and transparency in reporting. For further information on Nature Portfolio policies, see our Editorial Policies and the Editorial Policy Checklist.

Statistics

For all statistical analyses, confirm that the following items are present in the figure legend, table legend, main text, or Methods section.

n/a	Confirmed	
[ ]	[x]	The exact sample size (*n*) for each experimental group/condition, given as a discrete number and unit of measurement
[ ]	[x]	A statement on whether measurements were taken from distinct samples or whether the same sample was measured repeatedly
[ ]	[x]	The statistical test(s) used AND whether they are one- or two-sided<br>*Only common tests should be described solely by name; describe more complex techniques in the Methods section.*
[x]	[ ]	A description of all covariates tested
[x]	[ ]	A description of any assumptions or corrections, such as tests of normality and adjustment for multiple comparisons
[ ]	[x]	A full description of the statistical parameters including central tendency (e.g. means) or other basic estimates (e.g. regression coefficient) AND variation (e.g. standard deviation) or associated estimates of uncertainty (e.g. confidence intervals)
[ ]	[x]	For null hypothesis testing, the test statistic (e.g. *F*, *t*, *r*) with confidence intervals, effect sizes, degrees of freedom and *P* value noted<br>*Give P values as exact values whenever suitable.*
[x]	[ ]	For Bayesian analysis, information on the choice of priors and Markov chain Monte Carlo settings
[x]	[ ]	For hierarchical and complex designs, identification of the appropriate level for tests and full reporting of outcomes
[ ]	[x]	Estimates of effect sizes (e.g. Cohen's *d*, Pearson's *r*), indicating how they were calculated

Our web collection on statistics for biologists contains articles on many of the points above.

Software and code

Policy information about availability of computer code

Data collection	Zeiss Zen Black v3 was used to acquire immunofluorescence images; Med Associates MEDPC 4 was used to acquire Pavlovian conditioning data; Inscopix nvoke and UCLA miniscope were used in-vivo calcium imaging collection; electrophysiology data was collected by Multiclamp 700B and a Digidata 1550B digitizer (Molecular devices);
Data analysis	electrophysiology data were analyzed using pClamp 10.6 software from Molecular Devices; The raw fluorescence images were analyzed by NIH, ImageJ 1.53 and Inscopix, IDEAS platform; Microsoft Excel Office 365 version was used to organize/compile data ; GraphPad Prism v7 & v9 were used to graph and perform summarized statistical analysis ; custom codes for in-vivo calcium image analysis were developed on MATLAB 2018b and tested on MacOS and Windows; behavioral data from MEDPC 4 was converted by custom script on Python v3.8.

For manuscripts utilizing custom algorithms or software that are central to the research but not yet described in published literature, software must be made available to editors and reviewers. We strongly encourage code deposition in a community repository (e.g. GitHub). See the Nature Portfolio guidelines for submitting code & software for further information.

Data

Policy information about availability of data

All manuscripts must include a data availability statement. This statement should provide the following information, where applicable:

- Accession codes, unique identifiers, or web links for publicly available datasets
- A description of any restrictions on data availability
- For clinical datasets or third party data, please ensure that the statement adheres to our policy

Raw data can be found in Source Data file provided with this paper.

Research involving human participants, their data, or biological material

Policy information about studies with human participants or human data. See also policy information about sex, gender (identity/presentation), and sexual orientation and race, ethnicity and racism.

Reporting on sex and gender	N/A
Reporting on race, ethnicity, or other socially relevant groupings	N/A
Population characteristics	N/A
Recruitment	N/A
Ethics oversight	N/A

Note that full information on the approval of the study protocol must also be provided in the manuscript.

Field-specific reporting

Please select the one below that is the best fit for your research. If you are not sure, read the appropriate sections before making your selection.

☒ Life sciences ☐ Behavioural & social sciences ☐ Ecological, evolutionary & environmental sciences

For a reference copy of the document with all sections, see nature.com/documents/nr-reporting-summary-flat.pdf

Life sciences study design

All studies must disclose on these points even when the disclosure is negative.

Sample size	The number of animals used for the in vivo imaging experiment and behavioral tests was based on prior studies involving in vivo calcium imaging with motor-learning behavior components, and aligned with sample sizes reported in the literature. The number of cells included in the statistical analysis was determined using a similar approach.
Data exclusions	During the calcium imaging experiments, data were excluded if video files were corrupted during acquisition or if animals experienced severe complications at the surgical site.
Replication	calcium imaging experiments and analysis were first performed by using UCLA miniscope and then replicated by using Inscopix nVoke system. Also the original experiments were done using LHX6-cre strain (the data is not presented in this paper) which dominantly labeling SST+ cells in the motor cortex L5, then we replicated the results using SST-Cre strain in this paper.
Randomization	Animals were randomly assigned to experimental groups.
Blinding	Chemogenetic and optogenetic animal preparation were conducted in a blinded manner, and the data were separated during the statistical analysis. Calcium imaging experiment does not involve comparison between control and treatment groups.

Reporting for specific materials, systems and methods

We require information from authors about some types of materials, experimental systems and methods used in many studies. Here, indicate whether each material, system or method listed is relevant to your study. If you are not sure if a list item applies to your research, read the appropriate section before selecting a response.

Materials & experimental systems

n/a	Involved in the study	
☐	☒	Antibodies
☒	☐	Eukaryotic cell lines
☒	☐	Palaeontology and archaeology
☐	☒	Animals and other organisms
☒	☐	Clinical data
☒	☐	Dual use research of concern
☒	☐	Plants

Methods

n/a	Involved in the study	
☒	☐	ChIP-seq
☒	☐	Flow cytometry
☐	☐	MRI-based neuroimaging

Antibodies

Antibodies used	Rabbit anti-DsRed (Takara, #632496) or Chicken Polyclonal anti-GFP antibody (Abcam, #ab13970), Goat anti Rabbit-AlexaFluor568 (Invitrogen, A-11011) or Goat anti-chicken AlexaFlour 488(Invitrogen, #A-11039), DAPI Fluoromount-G (SouthernBiotech, 0100-20).
Validation	Each antibody is commercially available has been validated previously in the lab, by other groups, and/or by the respective manufacturers. Research Resource Identifiers (RRIDs) are listed for each antibody.<br>https://www.sigmaaldrich.com/US/en/product/mm/mab318<br>https://nittobo-nmd.co.jp/pdf/reagents/GFP.pdf

Animals and other research organisms

Policy information about studies involving animals; ARRIVE guidelines recommended for reporting animal research, and Sex and Gender in Research

Laboratory animals	Wild-type C57/Bl6J mice (JAX Stock #: 000664) were purchased from the Jackson Laboratory. Homozygous SST-Cre mice (JAX stock #: 013044; Sst tm2.1(cre)Zjh /J) were crossed with the wild-type C57BL6/J mice (JAX Stock #: 000664)
Wild animals	No wild animals were used in this study.
Reporting on sex	Both male and female mice were used in each genetic line and all experiments
Field-collected samples	No field-collected samples were used in this study.
Ethics oversight	All procedures carried out in this study were in accordance with the NIH Guidelines for the Care and Use of Laboratory Animals and were approved by the Animal Care and Use Committee of the National Institute on Alcohol Abuse and Alcoholism.

Note that full information on the approval of the study protocol must also be provided in the manuscript.

Plants

Seed stocks	N/A
Novel plant genotypes	N/A
Authentication	N/A

Magnetic resonance imaging

Experimental design

Design type	N/A
Design specifications	N/A
Behavioral performance measures	N/A

Acquisition

Imaging type(s)	N/A
Field strength	N/A
Sequence & imaging parameters	N/A
Area of acquisition	N/A

Diffusion MRI ☐ Used ☐ Not used

Preprocessing

Preprocessing software	N/A
Normalization	N/A
Normalization template	N/A
Noise and artifact removal	N/A
Volume censoring	N/A

Statistical modeling & inference

Model type and settings	N/A
Effect(s) tested	N/A

Specify type of analysis: ☐ Whole brain ☐ ROI-based ☐ Both

Statistic type for inference (See Eklund et al. 2016)	N/A
Correction	N/A

Models & analysis

n/a	Involved in the study
☐	☐ Functional and/or effective connectivity
☐	☐ Graph analysis
☐	☐ Multivariate modeling or predictive analysis

Functional and/or effective connectivity	N/A
Graph analysis	N/A
Multivariate modeling and predictive analysis	N/A
